# Optimising the outcomes of index admission laparoscopic cholecystectomy and bile duct exploration for biliary emergencies: a service model

**DOI:** 10.1007/s00464-020-07900-1

**Published:** 2020-08-28

**Authors:** Ahmad H. M. Nassar, Hwei J. Ng, Zubir Ahmed, Arkadiusz Peter Wysocki, Colin Wood, Ayman Abdellatif

**Affiliations:** 1 Laparoscopic Biliary Service, University Hospital Monklands, Airdrie, Scotland UK; 2 NHS Greater Glasgow and Clyde, Glasgow, UK; 3 Logan Hospital, Corner Meadowbrook and Loganlea Roads, Meadowbrook, QLD 4133 Australia

**Keywords:** Laparoscopic cholecystectomy, Emergency surgery, Biliary emergencies, Gall stones, Index admission surgery, Difficulty grading, Nassar Scale

## Abstract

**Aims:**

The rate of acute laparoscopic cholecystectomy remains low due to operational constraints. The purpose of this study is to evaluate a service model of index admission cholecystectomy with referral protocols, refined logistics and targeted job planning.

**Methods:**

A prospectively maintained dataset was evaluated to determine the processes of care and outcomes of patients undergoing emergency biliary surgery. The lead author has maintained a 28 years prospective database capturing standard demographic data, intraoperative details including the difficulty of cholecystectomy as well as postoperative outcome parameters and follow up data.

**Results:**

Over five thousand (5555) consecutive laparoscopic cholecystectomies were performed. Only patients undergoing emergency procedures (2399,43.2% of entire group) were analysed for this study. The median age was 52 years with 70% being female. The majority were admitted with biliary pain (34%), obstructive jaundice (26%) and acute cholecystitis (16%). 63% were referred by other surgeons. 80% underwent surgery within 5 days (40% within 24 h). Cholecystectomies were performed on scheduled lists (44%) or dedicated emergency lists (29%). Two thirds had suspected bile duct stones and 38.1% underwent bile duct exploration. The median operating time was 75 min, median hospital stay 7 days, conversion rate 0.8%, morbidity 8.9% and mortality rate 0.2%.

**Conclusion:**

Index admission cholecystectomy for biliary emergencies can have low rates of morbidity and mortality. Timely referral and flexible theatre lists facilitate the service, optimising clinical results, number of biliary episodes, hospital stay and presentation to resolution intervals. Cost benefits and reduced interval readmissions need to be weighed against the length of hospital stay per episode.

Gallstone related admissions represent nearly one third of emergency general surgery admissions in the United Kingdom—e.g. 15,000 in England in 2013–2014 [1]. Urgent cholecystectomy rates (within 10 days of first admission) for acute cholecystitis range from 0.2 to 35% across England [2]. Sinha et al. [3] and Harrison et al. [4] reported wide variations in the management and outcomes of cholecystectomy in England and Scotland respectively. This led to the establishment of National Institute for Health and Care Excellence (NICE) guideline on the management of gallstone diseases [5]. In part, the guideline recommends early laparoscopic cholecystectomy (within 1 week of diagnosis) in the setting of acute cholecystitis and surgical bile duct clearance at the time of cholecystectomy. However, the rate of early laparoscopic cholecystectomy remains low partly due to logistic and financial constraints [5, 6]. Shabanzadeh et al. [7] reported some 20% of patients with incidentally diagnosed gallstones will eventually develop symptoms or complications including acute cholecystitis, obstructive jaundice, acute cholangitis and acute pancreatitis. While guidelines are specific to the individual biliary emergencies [2, 8–14], these presentations occasionally overlap which may result in over-investigation and delays in non-specialist units.

The aim of this study was to evaluate a service model of index admission cholecystectomy including implementation and potential benefits of maximising index admission laparoscopic cholecystectomy and optimising resource utilisation.

## Methods

This is a cohort study of consecutive patients undergoing surgery for gallstone emergencies performed or directly supervised by a single surgeon between February 1992 and July 2019. The surgeon’s prospectively maintained laparoscopic cholecystectomy (LC) database was interrogated for patient demographics, admission presentation, previous biliary admissions, radiological findings and intervals from admission to referral and from referral to surgery. Additional studied parameters: American Society of Anaesthesiologists (ASA) classification, grade of operating surgeon, operative difficulty grade, operative time, conversion to open, perioperative complications, readmissions, number of episodes (total episodes including previous and current episode and any readmissions), number of weeks from presentation to resolution and mortality. The operative difficulty grade was based on the modified five grade Nassar Scale [15].

IRB approval was not required as the management protocols were consistent with the recommendations of national and international societies.

### Referral pathway

All emergency admissions with a clinical presentation suggestive of a biliary origin underwent abdominal ultrasound scanning (USS) and chest radiography (CXR). When calcular biliary pathology was confirmed patients were referred to the dedicated biliary team during the index admission. Referrals were accepted from the on-call surgical teams, from local physicians and occasionally from other hospitals for patients requiring laparoscopic bile duct exploration after failed ERCP. The referral protocol included mainly patients with suspected bile duct stones for the first five years of this series. It was established as a hospital protocol for managing all biliary emergencies by a specific firm/consultant as described above in 1997.

Informed consent was obtained from all patients with specific emphasis on the specialist nature of the unit about alternatives for the management of suspected bile duct stones. Patients fit for general anaesthesia (GA) proceeded directly to index admission LC with routine intraoperative cholangiography (IOC) and common bile duct exploration (CBDE) when indicated. Patients unfit for GA were managed conservatively or medically optimised to facilitate interval LC.

### Preoperative imaging

A minority of patients were referred by medical or external firms having already had cross-sectional imaging. Magnetic resonance chlaongio-pancreatography (MRCP) and computerised tomography scans of the abdomen and pelvis (CT AP) were only performed in patients with a high suspicion of malignancy or severe acute pancreatitis. Patients with severe cholecystitis and suspected gallbladder perforation associated with sepsis and rendering the patient unfit for surgery underwent CTAP on an intention to treat basis for potential radiologically guided percutaneous drainage.

### Job planning

The job plan of the consultant delivering the service was designed to allow index admission biliary surgery for a minimum emergency workload of 60%. This includes flexible programmed activities (PAs) for the biliary service and fewer outpatient clinics (two per month). This allowed the prompt preoperative assessment of patients admitted acutely and maximal utilisation of rapid access theatre lists.

### Theatre utilisation

Emergency biliary surgery was given priority (booked at short notice) on the surgeon’s scheduled lists, one slot was reserved for elective LC. Confidential enquiry into perioperative deaths (CEPOD) and emergency lists were also utilised. No lists which fell during the biliary team’s on-call periods were cancelled in keeping with the generic job plan.

### Operative techniques

A standard four-port technique, in the American position, with modified open access, was used. A blunt Duck Bill forceps (Karl Storz, Tuttlingen, Germany) was used for the dissection of the cystic pedicle, displaying the critical view of safety where feasible, and for separating the gall bladder from the cystic plate. Diathermy hook was not used in any cases during this study. Swab dissection or other blunt dissection was used when dense inflammatory adhesions were encountered. IOC was performed routinely (radiography staff and a dedicated image intensifier were available). Emergency laparoscopic cholecystectomies during weekends e.g. septic patients with acute cholecystitis were only occasionally necessary. The cystic duct and artery were secured with intracorporeal ties, occasionally using endoloops or sutures for wide cystic ducts.

As index admission cholecystectomies are associated with a significant percentage of acute cholecystitis and gallbladder empyema adopting optimal dissection techniques and adapting the selection of instruments are necessary to avoid complications and maintain a low conversion rate. Decompression of a distended gallbladder will facilitate the grasping and retraction of a thick walled gallbladder. Blunt swab or hydro dissection of dense inflammatory adhesions or the utilisation of a subserosal approach over Hartman’s pouch or body of the gallbladder to ensure dissection close to the gallbladder wall allows safe dissection of the cystic pedicle, particularly when displaying the critical view of safety is judged difficult or impossible. In such cases we attempt identifying the cystic artery lymph node as a safety marker, removing stones in the Hartman’s pouch or cystic duct and may resort to fundus first dissection. Once the fundus and the body of the gallbladder were freed blunt dissection may be facilitated by using the funnel technique; dividing the Hartman’s pouch around its circumference for posterior access. Transvesical access and cholangiography will confirm the integrity of the bile ducts and can allow further dissection and progress towards a complete cholecystectomy. Rarely, a fenestrated subtotal cholecystectomy was performed (0.1%) to avoid bile leakage from the stump, exclude bile duct stones and reduce the risk of residual or recurrent stones in the gallbladder remnant [16]. Techniques for safely dealing with cases of Mirizzi Syndrome have been described [17].

### Choledocholithiasis

The management of bile duct stones upon transition from open to LC in 1992 remained surgical and did not involve endoscopic preoperative clearance of the bile duct stones. Patients who were fit for anaesthesia continued to undergo IOC and bile duct exploration when indicated. Most patients initially had duct explorations through a choledochotomy. However, within a short period the choledochotomy approach was reserved for large, multiple or proximal CBD stones and transcystic exploration techniques were introduced and increasingly utilised. Blind basket exploration is succesful in a large proportion of cases where a few, distal and small stones are encountered [18, 19]. While choledochoscopy is used for all choledochotomy explorations it is reserved for transcystic explorations involving multiple, impacted or proximal stones [20].

### Postoperative management

The postoperative care of the most patients following emergency LC/bile duct explorations took place at ward level. Those with biliary drains (defined below) underwent tube cholangiography within a few days, before discharge, returning to the surgical ward two weeks postoperatively for removal. Follow up was conducted within three months at the outpatient clinic or, in recent years, via a telephone consultation. Those undergoing bile duct exploration were reviewed in the outpatient clinic within three to four months and annually afterwards to detect any related readmissions and monitor the rate of CBD stone recurrence.

## Results

Of 5555 LC performed between February 1992 and July 2019, 2399 (43.2%) were emergency admissions. Most of the patients were female (70.1%) and the median age was 53 years (13–91). ASA classification was mostly 1 to 3 (35.3%, 42.9% and 17.8% respectively).

The primary diagnoses in this series included 819 (34.1%) biliary colic, 615 (25.6%) obstructive jaundice, 392 (16.3%) acute cholecystitis, 220 (9.2%) acute gallstone pancreatitis, 177 (7.4%) jaundice with acute pancreatitis, 95 (4.0%) jaundice with acute cholangitis, 66 (2.8%) jaundice with acute cholecystitis and 15 (0.6%) acute cholangitis.

The main source of referral was other surgeons *n* = 1519 (63.3%). A quarter was admitted directly under the care of the biliary team whilst on-call. The remainder were from other hospitals (*n* = 183, 7.6%) or local physicians (*n* = 145, 6.0%).

Previous biliary admissions were recorded in 495 patients (20.6%) more than a quarter (28.5%) were to other hospitals (*N* = 141). Very few (*N* = 44; 1.8%) were previously unfit for LC and were treated conservatively by the biliary firm and optimised for delayed cholecystectomy.

Preoperative USS was recorded in 2232 patients (93.0%). MRCP was requested in 202 (8.4%) and CT AP in 166 (6.9%) patients. Preoperative ERCP was done in 115 patients (4.8%), the majority at source institutions where failed ERCP or endoscopic CBD clearance prompted the referral to the biliary team. Based on clinical, biochemical or radiological criteria, 1572 patients (65.5%) had risk factors for bile duct stones. This high percentage reflected the nature of external referrals attributed to the interest of this biliary firm in single session management of bile duct stones.

**Fig. 1:**
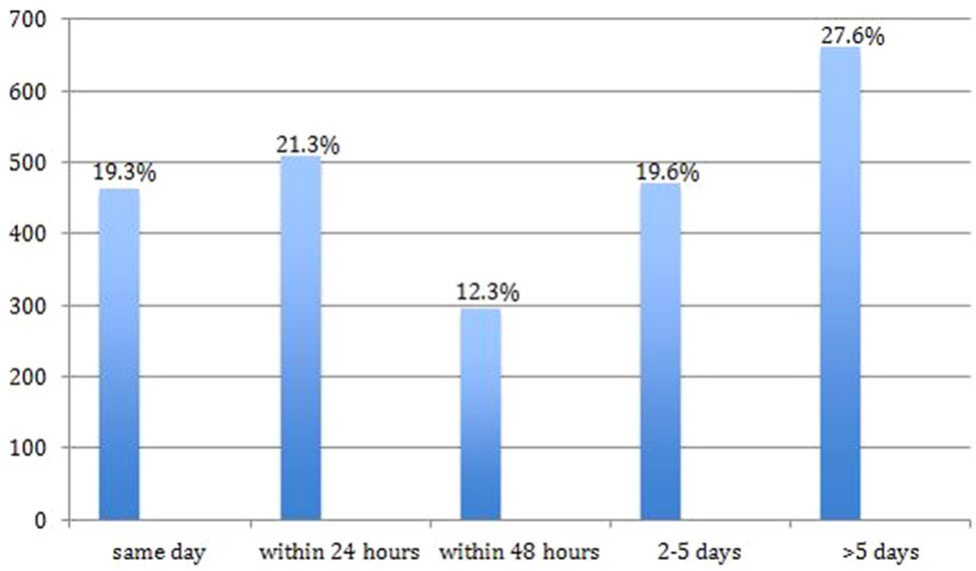
Timing from admission with an acute biliary presentation to laparoscopic cholecystectomy

The interval from admission to LC is shown on Fig. 1. 80% of patients (1929 patients) underwent LC within 5 days of referral. The type of operating list utilised was known for 1655 patients (this parameter was not recorded prior to 2003). 727 LC (43.9%) were performed on scheduled lists, 485 (29.3%) in a dedicated CEPOD theatre, 433 (26.2%) while on-call and 10 (0.6%) on ad-hoc lists.

The LC was considered difficult in 47.8% (Grades III to V on the modified Nassar Difficulty Scale [15]) as shown in Table 1. Division of adhesions between the gallbladder and the duodenum or hepatic flexure was necessary in 631 (26.3%). The cystic pedicle was judged difficult in 1232 cases (51.4%) and the cystic duct was wide in 475 cases (19.8%) and contained stones in 507 (21.1%). An operative diagnosis of acute cholecystitis or empyema was made in 570 cases (24.3%). Twenty-four (1.0%) cholecystoenteric fistulae were encountered while fundus first dissection was performed in 87 cases (3.6%).

**Table 1:** Preoperative data and operative parameters of patients undergoing laparoscopic cholecystectomy for acute biliary presentations

Perioperative characteristics	Patients (*n* = 2399) (%)
Source of referral	
Other surgical consultant	1519 (63.3%)
Own biliary team on-call	552 (23.1%)
Other hospitals	183 (7.6%)
Physicians	145 (6.0%)
Number of previous admissions (*n* = 495)	
1	458 (19.1%)
2	33 (1.4%)
3 or more	4 (0.1%)
Referral to surgery interval in days	
≤ 5	1929 (80.4%)
6–10	313 (13.0%)
≥ 11	100 (4.2%)
Not recorded	57 (2.4%)
Operative difficulty grade	
I	560 (23.3%)
II	673 (28.1%)
III	553 (23.1%)
IV	541 (22.6%)
V	68 (2.8%)
Not recorded	4 (0.2%)
Intra-operative cholangiography	
Yes	2343 (97.7%)
No	56 (2.3%)
Bile duct exploration	913 (38.1%)
Transcystic	578(24.1%)
Choledochotomy	335 (14.0%)
Operating time (median, range) minutes	75 (15–570)
Conversion rate	19 (0.8%)

Intraoperative cholangiography was performed successfully in 97.7% and showed abnormalities necessitating laparoscopic bile duct exploration in 913 cases (38.1%). Transcystic exploration was carried out in 63.3% and 36.6% via a choledochotomy. Choledochoscopy was utilised in 583 explorations (63.9%). Biliary drainage was established in 389 (42.6%) of all urgent explorations; using transcystic tube in 216 (23.7%), T-tube in 161 (17.6%) and biliary stent in 12 (1.3%). Primary closure of a choledochotomy was carried out in 21 cases (2.3%). The perioperative findings are summarised in Table 1.

The median operating time was 75 min (74 min for LC and IOC and 105 min for bile duct explorations) with 478 (19.9%) cases performed fully or partly by trainees. There were 19 conversions to open cholecystectomy (0.8%). 31.5% of the conversions (6 patients) occurred in the early part of the study and were due to impacted CBD stones resulting in the failure of laparoscopic bile duct exploration. Four conversions occurred in the setting of Mirizzi Syndrome, including two who underwent bilioenteric drainage.

The median total hospital stay was 7 (1–63) days, including awaiting imaging prior to referral, and the median presentation to resolution interval was 2 weeks (1–140 weeks). For the purpose of this study these outcome parameters included earlier admissions to other institutions ads shown in Table 2.

**Table 2:** Postoperative outcome parameter

Postoperative data and outcome parameters	Patients (*n* = 2399) (%)
Peri-operative complication rate	210 (8.8%)
Mortality rate	5 (0.2%)
Duration of total hospital stay (median, range) days^a^	7 (1–63)
Presentation to resolution period (median, range) weeks^a^	2 (1–140)
Number of admission episodes per patient^a^	1.3

^a^Including all pre-referral previous admissions at other institutions or units where applicable

The total morbidity rate was 8.9%, including 4.6% operative or perioperative complications and 4.3% complications leading to readmissions. The perioperative complications are shown in Table 3. The causes of 30-day readmissions are summarised in Table 4. Most complications (76.1%) were treated conservatively. Bile leakage occurred in 19/2399 cases (0.8%) leading to five readmissions. 14 of these had undergone a bile duct exploration (9 via choledochotomy): 8 settled conservatively, five needed ERCP and one a percutaneous drainage. Post cholecystectomy bile leaks occurred in five cases: three subvesical ducts were found at re-laparoscopy, one needed percutaneous drainage and one settled conservatively without confirmation of the site. All patients remained asymptomatic on follow up.

**Table 3:** Operative and perioperative complications

Peri-operative complications	Treatment		Total (*n* = 111)	Post operative hospital stay (mean) days	Clavien Dindo classification
	Conservative (*n* = 81)	Reintervention (*n* = 30)			
Small bowel perforation during adhesiolysis/port insertion	2 Primary closure 2 Resection	–	4	No record	G3b
Liver injury due to primary epigastric port	1	–	1	4.0	G2
Surgical emphysema	1	–	1	8.0	G1
Hypoxia	1	–	1	5.0	G3b
Post operative unstable angina	1	–	1	No record	G1
Blood transfusion	1	–	1	No record	G2
Shingles	1	–	1	8.0	G1
Post operative pyrexial of unknown origin	2	–	2	8.0	G2
Post operative pancreatitis	9	–	9	4.1	G1
Urinary retention	4	–	4	3.5	G1
Post operative myocardial infarction	1	–	1	9.0	G4
Post operative perforated duodenal ulcer	–	1 Re-laparoscopy	1	No record	G3b
Stroke/ TIA	4	–	4	No record	G2
Chest infection	10	2 Re-ventilation	12	4.3	G2, G3b
Post operative jaundice	4	1 ERCP	5	2.8	G1, G3a
Bile leak	10	4 ERCP/stenting	14	7.1	G3a
Retained stone	–	11 ERCP	11	2.9	G3a
Acute kidney injury secondary to T-tube loss	4	–	4	11.7	G1
Abdominal pain after removal of T-tube	3	–	3	14.0	G1
Retained T-tube/transcystic tube	1 Removed 2 weeks later	3 Re-laparoscopy 1 ERCP	5	19.2	G1, G3a, G3b
Blood clot in CBD dissolved with Alteplase	3	–	3	12.7	G2
Post operative collection	1	5 Percutaneous drainage	6	4.2	G3a
Failed ERCP stenting, Mirizzi Type II & III	–	1 Re-laparoscopy 1 Relaparotomy	2	2.0	G3b
Wound infection^a^	15	–	15	2.1	G1

*TIA* transient ischaemic attack, *CBD* common bile duct, *ERCP* endoscopic retrograde cholangiopancreatography

^a^Wound infections probably under reported as some are treated in the community and are not reported

**Table 4:** 30 days postoperative complications requiring readmission

Causes of 30-day post operative complications requiring readmission	Treatment		Number (*n* = 102)	Clavien Dindo classification
	Conservative (*n* = 81)	Reintervention (*n* = 21)		
Acute kidney injury due to T-tube fluid loss	10	–	10	G1
Dislodged/retained /retracted T-tube or Transcystic tube	–	2 Re-laparosocopy 2 Gastroscopy	4	G3a, G3b
Abdominal pain post T-tube/transcystic tube removal	18	–	18	G1
Bile leak	1	3 Re-laparoscopy 1 Percutaneous drainage	5	G1, G3a, G3b
Retained CBD stone	–	6 ERCP	6	G3a
Jaundice	1	2 ERCP (stent benign hepatic duct stricture and to unblock stent)	3	G1, G3a
Pancreatitis and sequelae	9	–	9	G1
Umbilical port haematoma	2	–	2	G1
Wound infection	2	–	2	G1
Sepsis	2	1 Liver failure requiring ITU support	3	G4
PE/ DVT	2	–	2	G1
Post operative collection	5	2 Percutaneous drainage	7	G3a
Non specific abdominal pain	26	–	26	G1
Diarrhoea	1	–	1	G1
Chest infection	1	–	1	G2
Urinary retention	1	–	1	G1
Gallbladder cancer/ pancreatic cancer	–	2 Biliary reconstruction	1	G3b

*CBD* common bile duct, *ERCP* endoscopic retrograde cholangiopancreatography, *ITU* intensive therapy unit, *PE* pulmonary embolus, *DVT* deep venous thrombosis

The total morbidity specific to bile duct exploration in this study was 5.8%. There were 44/913 complications related to biliary drainage (4.8%) and postoperative pancreatitis occurred in 1%. Reintervention was necessary in less than 1.8% (16/913). This would compare favourably with the published results of preoperative endoscopic clearance using ERCP.

Reinterventions were thus required in 51 patients (2.1%), including 25 ERCP, 10 re-laparoscopies, three re-laparotomies, eight image guided percutaneous drainage procedures, three re-ventilations and two upper GI endoscopies.

There were 5 (0.2%) deaths. One patient with a freely perforated gallbladder died from ongoing sepsis. Two elderly patients (ASA class 3) had uneventful bile duct explorations for Mirizzi Type 2 but died from severe postoperative pneumonia some three weeks later (operative difficulty grades were 4 and 5). The fourth patient died following attempted embolisation of an incidentally discovered abnormal left hepatic artery at a specialist unit. The fifth patient with peripheral vascular disease died of mesenteric ischemia and total small bowel infarction on post operative day 4.

## Discussion

A biliary service supported by a clear hospital-wide referral protocol, flexible job planning and operating lists is able to deliver safe early/index admission cholecystectomy for patients presenting acutely with a variety of gallstone complications in keeping with guidelines. [2, 8, 9, 11, 21]. MRCP (8.4%) and abdominal CT (6.9%) were infrequently required as was preoperative ERCP (4.8%). Four out of five patients (80.4%) underwent cholecystectomy within 5 days of referral. Routine IOC and a high bile duct exploration rate resulted in a very low rate of retained stones or unresolved jaundice (*N* = 25; 1.0%). Operative difficulty was relatively evenly distributed between Nassar difficulty grades I and IV (one quarter each). Three quarters of all complications were managed conservatively (162/210; 77.1%).

The CholeS study [22] is now widely seen as providing a contemporary snapshot of cholecystectomy practice in the UK. In comparison, the current study demonstrates greater numbers of LCs performed as emergencies (43.2% v 16.3%) with much lower use of cross-sectional imaging—MRCP and CT use was over threefold lower than national data (28.9% vs 8.4%) and (20.1% vs 6.9%) respectively [23]. The rate of previous emergency biliary admission in this study was 9% (495/5555 patients) compared to 37% in the national study. We have previously demonstrated a strong association between previous biliary admissions and increased technical difficulty of cholecystectomy. [24].

IOC and management of choledocholithiasis remain controversial. Two session management of bile duct stones (ERCP followed by LC) is the standard treatment in many centres. The most recent meta-analysis showed pre-cholecystectomy ERCP had a higher rate of stone clearance rate (OR 1.63; 95% CI 1.16–2.28; *p* = 0.005) and lower rate of bile leaks (OR 4.09; 95% CI 2.09–8.01; *p* < 0.0001) balanced against a higher rate of pancreatitis (OR 0.23; 95% CI 0.11–0.50; *p* = 0.0002) and longer length of hospital stay (– 2.46 days; 95% CI – 3.67 to – 1.24; *p* < 0.0001) when compared to laparoscopic bile duct exploration [25].

Because this unit continued to view choledocholithiasis as a surgical condition upon transition from open to LC in 1992, IOC remained the definitive form of bile duct imaging followed by laparoscopic bile duct exploration where indicated. This approach was reinforced by good outcomes and justified by cost benefits of index management of the entire episode of care stemming from minimal use of preoperative imaging and a shorter length of stay. The NICE guidelines costing statement [26] suggested that “using the national tariff 2014–2015 the weighted average cost of clearing the bile duct with endoscopic ERCP before LC is estimated at £1607”. The use of ERCP in the emergency cohort was only 4.8% with the majority being failed ERCP referrals from other centres and patients unfit for anaesthesia. Preoperative ERCP was initiated by this service in 2.9% (*n* = 65/2253) while the CholeS [22] cited a national rate of preoperative ERCP of 9.6%. This suggests that, if laparoscopic bile duct exploration was widely performed, the demand on ERCP services nationwide can be reduced threefold.

The incidences of 30 days readmissions, 30 day complications and 30 days reinterventions were all lower than the CholeS national study. Although the total hospital stay in our study was longer, we included all pre-referral episodes at other departments and hospitals. The low morbidity specific to bile duct exploration in this study (1% pancreatitis and 1.8% reintervention) compares favourably with preoperative endoscopic clearance using ERCP (4.2% pancreatitis) and 5.8% bile leak following LCBDE [25].

Concerns persist among surgeons that early LC for acute cholecystitis has higher morbidity and conversion rates than delayed LC but this is contrary to available evidence and guidelines [5, 27, 28] and is not supported by the data of the current study. The safety and cost efficiency of emergency LC has been demonstrated [29, 30]. Khan et al. [31] concluded that the cost savings of early LC in district hospitals could be used to establish dedicated hot gallbladder lists. Bokhari et al. [27] suggested emergency cholecystectomy lists similar to our service model without the additional provision of managing bile duct stones.

Cooperation with other surgical firms and hospital departments facilitates the streamlining of patient transfer and scheduling. The radiology department plays an important role when occasional CT or MRCP are requested on an urgent basis in order to expedite the scheduling of surgery. Radiographer support for IOC has been optimised and helps to minimise the duration of surgery. The contribution of the radiographers and the optimisation of their involvement and the technical aspects of cholangiography have previously been reported by this unit [32, 33].

Possible limitations of this service model include the prioritisation of access to emergency theatre and the availability of personnel trained in emergency biliary surgery. The full range of the service may not be applicable to units without expertise in bile duct exploration.

## Conclusion

Index admission LC for biliary emergencies is feasible for most patients in a dedicated biliary unit. Minor adjustments to existing surgical service structures could result in optimising the utilisation of cross-sectional imaging and minimising re-presentations and hospital stay with added cost efficiency. This service model can still be implemented for most biliary emergencies even in units without the expertise or the facilities for single session management of bile duct stones.

Flexible consultant job planning, clear referral protocols and optimal theatre utilisation are vital components of an acute biliary service. And surgical skills can be refined over time.
